# The influence of different metal-chelate conjugates of pentixafor on the CXCR4 affinity

**DOI:** 10.1186/s13550-016-0193-8

**Published:** 2016-04-26

**Authors:** Andreas Poschenrieder, Margret Schottelius, Markus Schwaiger, Horst Kessler, Hans-Jürgen Wester

**Affiliations:** Pharmaceutical Radiochemistry, Technical University Munich, Walther-Meißner-Str.3, 85748 Garching, Germany; Department of Nuclear Medicine, Technical University Munich, Klinikum rechts der Isar, Ismaninger Straße 22, 81675 Munich, Germany; Institute for Advanced Study at the Department Chemie, Technical University Munich, Lichtenbergstr. 2a, 85748 Garching, Germany

**Keywords:** GPCR, CXCR4, [^68^Ga]pentixafor, Pentapeptide, DOTA, Chelator, Radiopharmaceutical, Tracer, Cancer

## Abstract

**Background:**

The overexpression of the chemokine receptor 4 (CXCR4) in different epithelial, mesenchymal, and hematopoietic cancers makes CXCR4 an attractive diagnostic and therapeutic target. However, targeting the CXCR4 receptor with small cyclic pentapeptide-based radiopharmaceuticals remains challenging because minor structural modifications within the ligand-linker-chelate structure often significantly affect the receptor affinity. Based on the excellent in vivo properties of CXCR4-directed pentapeptide [^68^Ga]pentixafor (cyclo(-d-Tyr-*N*-Me-d-Orn(AMB-DOTA)-l-Arg-l-2-Nal-Gly-)), this study aims to broaden the spectrum of applicable (radio)metal-labeled pentixafor analogs.

**Methods:**

Cyclic pentapeptides, based on the pentixafor scaffold, were synthesized by a combined solid- and solution-phase peptide synthesis. The CXCR4 receptor affinities of the cold reference compounds were determined in competitive binding assays using CXCR4-expressing Jurkat T - cell leukemia cells and [^125^I]FC131 as the radioligand.

**Results:**

Metalated pentixafor derivatives with cyclic and acyclic chelators were synthesized by solid-phase peptide synthesis and evaluated in vitro. The resulting CXCR4 affinities (IC_50_) were highly dependent on the chelator and metal used. Two pentapeptides, Ga-NOTA and Bi-DOTA conjugates, offer an improved affinity compared to [^68^Ga]pentixafor.

**Conclusions:**

Based on the pentapeptide [^68^Ga]pentixafor, a broad range of metal-labeled analogs were investigated. The affinities of the new compounds were found to be strongly dependent on both the chelator and the metal used. Bi-labeled pentixafor showed high receptor affinity and seems to be a promising ligand for further preclinical evaluation and future α-emitter-based endoradiotherapy.

## Background

**Fig. 1:**
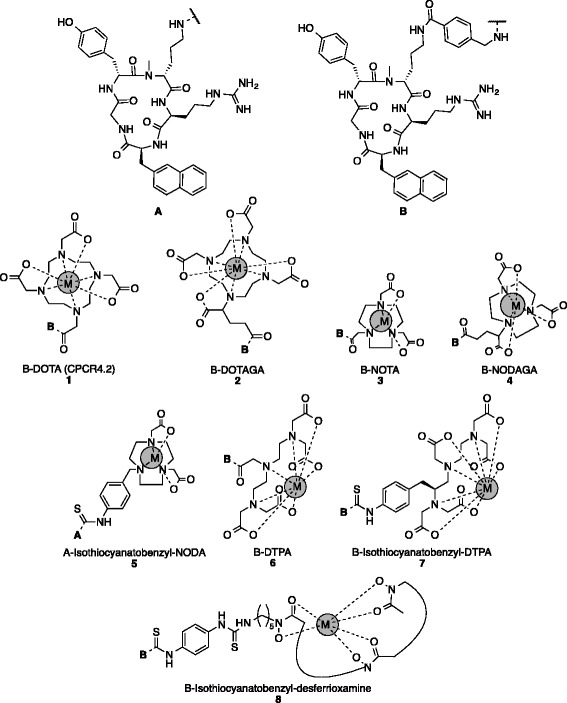
Pentixafor-based analogs with different chelators and metals (*M*, *gray*). **A** constitutes the pentapeptide core *cyclo(-* d *-Tyr-N-Me-* d *-Orn-* *l* *-Arg-* *l* *-2-Nal-Gly-)* whereas **B** contains an additional aminomethylbenzoyl (AMB) linker between the peptide and chelator

The chemokine receptor 4 (CXCR4) and its sole known natural ligand stromal cell derived factor-1 (SDF-1, CXCL12) are physiologically involved in leukocyte recruitment, homing, and retention of hematopoietic stem and progenitor cells [1, 2]. CXCR4 also holds a substantial role in various pathological conditions and represents a highly attractive therapeutic target. Overexpression of CXCR4 has been linked to cancer proliferation, cell migration, and tissue-specific homing of cancer cells as well as resistance to conventional and targeted therapies [3–5]. After the identification of CXCR4 as a co-receptor for the entry of the HIV virus [6], great efforts have been made towards the development of CXCR4 antagonists. Recently, our group has developed [^68^Ga]pentixafor (*cyclo(-*d*-Tyr-N-Me-*d*-Orn(*aminomethylbenzoyl (AMB)*-DOTA)-l-Arg-l-2-Nal-Gly-*), [^68^Ga]**1**, Fig. 1*)*, a cyclic pentapeptide with 5 nM affinity and high selectivity towards hCXCR4 [7, 8]. [^68^Ga]pentixafor exhibits high uptake and long retention in CXCR4-expressing tissues (6.16 % ± 1.16 % ID/g, 1 h post-injection (p.i.) and 4.63 % ± 1.54 %, 2 h p.i. in OH1 human small cell lung cancer tumor-bearing mice) and is rapidly cleared from non-target tissue (1.08 % ± 0.27 % in the blood, 1 h p.i.) and renally excreted [7]; the corresponding tumor-to-blood and tumor-to-muscle ratios are substantially higher than those compared to other peptidic CXCR4 imaging agents [8]. First, studies in patients suffering from lymphoproliferative diseases [9, 10] demonstrated its excellent properties, including a favorable dosimetry for CXCR4-receptor mapping by means of positron emission tomography (PET) [11].

Based on the success of [^68^Ga]pentixafor, this study aims to broaden the potential spectrum of radiometal-labeled CXCR4 ligands, both for imaging and therapy, by development and in vitro evaluation of a range of tracers labeled with alternative radionuclides, such as ^111^In^3+^ (for single photon emission computed tomography (SPECT)); ^18^F^−^ and ^89^Zr^4+^ (for PET); or ^90^Y^3+^, ^177^Lu^3+^, and ^213^Bi^3+^ (for endoradiotherapy).

However, the experiences gained during the development of pentixafor have shown that, compared with [^68^Ga]pentixafor, unlabeled pentixafor and other radiometalated pentixafor derivatives exhibit significantly lower CXCR4 receptor affinities. Thus, in contrast to other peptides, such as somatostatin receptor (SSTR), gastrin-releasing peptide receptor (GRPR), or α_v_β_3_ binding peptides, the affinity of [^68^Ga]pentixafor towards CXCR4 is determined by the entire ligand-spacer-chelator-radiometal construct. Consequently, a more or less independent “bioactive substructure” or “pharmacophor” (e.g., the pentapeptide core **A** depicted in Fig. 1) cannot be identified. In this study, we investigated pentixafor derivatives with alternative cyclic and acyclic chelators and evaluated these ligands in vitro. With regard to the utilized chelators, the following nuclides relevant for medical purposes have been investigated: Ga^3+^, AlF^2+^, Zr^4+^, Cu^2+^, In^3+^, Lu^3+^, Y^3+^, and Bi^3+^ (Fig. 1).

## Methods

### General

Trityl chloride polystyrene (TCP) resins were purchased from PepChem (Tübingen, Germany) and Sigma-Aldrich (Steinheim, Germany). 9-fluorenylmethyloxycarbonyl (Fmoc) and all other protected amino acid analogs were obtained from Iris Biotech (Marktredwitz, Germany) or Bachem (Bubendorf, Switzerland). Chelators were obtained from CheMatech (Dijon, France, or Macrocyclics (Dallas, USA)) while all other chemicals were bought from Sigma-Aldrich, Fluka, or Merck (Darmstadt, Germany) if not stated otherwise. Solvents and all other organic reagents were purchased from Sigma-Aldrich (Munich, Germany), CLN (Freising, Germany), and VWR (Darmstadt, Deutschland). Water for reversed phase (RP)-HPLC was filtered through a 0.2-μm filter (Thermo Scientific, Barnstead Smart2Pure, Niederelbert, Germany). Analytical RP-HPLC was performed on a Nucleosil 100 C18 (5 μm, 125 × 4.0 mm^2^) column (CS GmbH, Langerwehe, Germany) using a Sykam gradient HPLC System (Sykam GmbH, Eresing, Germany). For elution, linear gradients of acetonitrile (0.1 % (*v*/*v*) trifluoroacetic acid (TFA), solvent B) in water (0.1 % TFA (*v*/*v*), solvent A) at a constant flow of 1 mL/min over 15 min were used. UV detection was performed at 220 and 254 nm using a 206 PHD UV-vis detector (LinearTM Instruments Corporation, Reno, USA). Purities were determined at 220 nm using LabSolutions software by Shimadzu Corp. Preparative RP-HPLC was performed on a Sykam gradient HPLC System (Sykam GmbH, Eresing, Germany) equipped with a Multospher 100 RP 18-5 (250 × 20 mm^2^) column (CS GmbH, Langerwehe, Germany) at a constant flow of 10 mL/min using the same solvents as stated above (duration of gradient, 20 min). Electrospray ionization (ESI)-mass spectra were recorded on a Varian 500-MS IT mass spectrometer (Agilent Technologies, Santa Clara, USA).

#### General SPPS

Peptides were synthesized manually on solid TCP support using standard Fmoc strategy and an Intelli-Mixer syringe shaker (Neolab, Heidelberg, Germany). As side chain protecting groups, N-[1-(4,4-dimethyl-2,6-dioxocyclohexylidene)ethyl] (Dde) for d-Orn and t-butyl and 2,2,4,6,7-pentamethyldihydrobenzofuran-5-sulfonyl (Pbf) groups for d-Tyr and Arg, respectively, were utilized.

O-(Benzotriazol-1-yl)-N,N,N′,N′-tetramethyluronium tetrafluoroborate (TBTU) and N-hydroxybenzotriazole (HOBt) were used as coupling reagents. N-alkylated amines were acylated using (1-[bis(dimethylamino)methylene]-1H-1,2,3-triazolo[4,5-b]pyridinium 3-oxid hexafluorophosphate) (HATU) with 1-hydroxy-7-azabenzotriazole (HOAt) as racemization suppressant.

Cleavage of the peptide from the TCP support with retention of acid-labile protection groups was achieved by treating the peptidyl resin with a solution of dichloromethane (DCM):trifluoroethanol:acetic acid (6:1:3) (*v*/*v*/*v*); for deprotection of acid-labile groups, TFA:triisopropylsilane (TIPS):H_2_O (95:2.5:2.5) (*v*/*v*/*v*) was used (2× 30 min). Deprotection of Dde was carried out with 2 % hydrazine monohydrate in dimethylformamide (DMF) (*v*/*v*).

For Fmoc-deprotection, the peptide was treated with 20 % piperidine in N-methyl-2-pyrrolidone (NMP) (*v*/*v*) for 20 min.

For general coupling of amino acids, a solution of Fmoc-Xaa-OH (1.5 equiv), TBTU (1.5 equiv), HOBt (1.5 equiv), and N,N-diisopropylethylamine (DIPEA) (5 equiv) in NMP (1 mL/g resin) was added to the resin-bound peptide and shaken for 90 min at room temperature and washed six times with NMP.

#### Synthesis of **A**

The peptide was synthesized according to a previously published procedure [12]. In short, synthesis was carried out using a standard Fmoc strategy using a TCP resin as solid support and HOBt/TBTU as coupling reagents. After selective *N-*methylation of d-Orn, d-Tyr was coupled to the peptide with HATU/HOAt, cleaved from the resin, and finally cyclized.

Coupling AMB to the d-Orn side chain was carried out using Fmoc- or Boc-protected AMB (1.5 equiv). AMB was preactivated with DIC (N,N′-diisopropyl-carbodiimide) (1.5 equiv), HOAt (1.5 equiv), and DIPEA (4.5 equiv) in 5 mL DMF for 10 min. The d-Orn deprotected peptide precursor was dissolved in DMF, and the preactivated linker was added. After complete reaction (2 h), Fmoc deprotection and semi-preparative HPLC purification, appropriate chelators were coupled to the peptide. HPLC (50 to 100 % B in 15 min): *t*_*R*_ = 8.0 min; ESI-mass spectra (MS): calculated for (C61H78N10O10S): 1142.5; found: *m/z* = 1143.6 [M+H]^+^.

#### Coupling of chelators and metal complexation

A molar excess of the activated chelator was added to a free amino group of the peptide analog. Subsequent to successful coupling, the chelator-conjugated peptide was deprotected and purified. Metal complexation was performed in the presence of weakly chelating acetate buffers to reduce the likelihood of hydrolysis. Solutions for metal labeling comprised LuCl_3_ (20 mM), pH = 6.0; InCl_3_ (20 mM), pH = 4.5; and YCl_3_ (20 mM), pH = 5.9, each in ammonium acetate (0.1 M) and Ga(NO_3_)_3_ (2 mM) pH = 3.0; Cu(OAc)_2_ pH = 6.0; and ZrCl_4_ (20 mM) pH = 1.3, each in water. The chelator-conjugated peptide (250 μL, 2 mM, 1 equiv) was dissolved in H_2_O and DMSO up to 50 % (*v*/*v*), if necessary, and the metal (1–10 equiv) was added, pH adjusted, and heated for 30 min. Final metalated peptides were obtained in a purity ≥ 95 %, and used for in vitro studies without further purification, unless stated otherwise.

#### Coupling of DOTA or NOTA

For DOTA/NOTA derivatization, 1,4,7,10-tetraazacyclododecane-1,4,7,10-tetraacetic acid (DOTA) or 1,4,7-triazacyclononane-triacetic acid (NOTA) (1 equiv) was preactivated for 20 min using N-hydroxysuccinimide (NHS) (1.25 equiv), 1-ethyl-3-(3-dimethylaminopropyl)carbodiimide (EDCI) (1.25 equiv), and DIPEA (2 equiv) dissolved in water (1 mL/0.3 mmol). The peptide **B** (0.25 or 0.3 equiv, for DOTA or NOTA, respectively) was dissolved in DMF (1 mL per 0.15 mmol of peptide) and slowly added to the reaction mixture according to a previously published protocol [13]. **3**: HPLC (30 to 55 % B in 15 min): *t*_*R*_ = 10.1 min; ESI-MS: calculated for (C56H73N13O12): 1119.6; found: *m/z* = 1121.0 [M+H]^+^, 1143.0 [M+Na]^+^; **1**: HPLC (30 to 55 % B in 15 min): *t*_*R*_ = 8.8 min; ESI-MS: calculated for (C60H80N14O14): 1220.6; found: *m/z* = 1221.6 [M+H]^+^, 1243.6 [M+Na]^+^.

#### Coupling of DOTAGA-anhydride

2,2′,2″-(10-(2,6-dioxotetrahydro-2H-pyran-3-yl)-1,4,7,10-tetraazacyclododecane-1,4,7-triyl)triacetic acid (DOTAGA-anhydride) (2 equiv) was dissolved in anhydrous DMF to obtain a white suspension and added to a solution of **B** (1 equiv) and triethylamine (10 equiv) in anhydrous DMF. **2**: HPLC (30 to 55 % B in 15 min): *t*_*R*_ = 9.8 min; ESI-MS: calculated for (C63H84N14O16): 1292.6; found: *m/z* = 1293.5 [M+H]^+^, 1315.5 [M+Na]^+^.

#### Coupling of NODAGA

4-(4,7-bis(2-(tert-butoxy)-2-oxoethyl)-1,4,7-triaza cyclononan-1-yl)-5-(tert-butoxy)-5-oxopentanoic acid ((NODAGA)(tBu)_3_) (3 equiv) was coupled to **B** (1 equiv) using a solution of DIC (2 equiv), HOAt (2 equiv), and DIPEA (6 equiv) in anhydrous DMF. The product was fully deprotected and purified. HPLC (30 to 55 % B in 15 min): *t*_*R*_ = 10.8 min; ESI-MS: calculated for (C59H77N13O14): 1191.6; found: *m/z* = 1193.0 [M+H]^+^, 1215.0 [M+Na]^+^.

#### Coupling of DTPA

3,6,9-tris(2-(tert-butoxy)-2-oxoethyl)-13,13-dimethyl-11-oxo-12-oxa-3,6,9-triazatetradecan-1-oic acid (DTPA)(tBu)_4_ (2 equiv) was coupled to **B** (1 equiv) using a solution of DIC (1.5 equiv), HOAt (1.5 equiv), and DIPEA (4.5 equiv) in anhydrous DMF. After successful coupling, the product was deprotected and purified. HPLC (30 to 60 % B in 15 min): *t*_*R*_ = 8.0 min; ESI-MS: calculated for (C58H75N13O16): 1209.6; found: *m/z* = 1210.8 [M+H]^+^, 1232.6 [M+Na]^+^.

#### Coupling of p-SCN-Bn-DTPA

2-(4-isothiocyanatobenzyl)diethylenetriaminepentaacetic acid (p-SCN-Bn-DTPA) (21.0 mg, 32.4 μmol, 1.5 equiv) was coupled to **B** (18.0 mg, 21.6 μmol, 1 equiv) with DIPEA in anhydrous DMF to obtain pH ≈ 9. HPLC (30 to 60 % B in 15 min): *t*_*R*_ = 11.2 min; ESI-MS: calculated for (C66H82N14O17S): 1374.6; found: *m/z* = 1375.8 [M+H]^+^.

#### Coupling of p-SCN-Bn-DFO

p-Isothiocyanatobenzyl-desferoxamine (DFO-Bn-NCS) (21.0 mg, 32.4 μmol, 1.5 equiv) was coupled to **B** (18.0 mg, 21.6 μmol, 1 equiv) in anhydrous DMF and adjusted with NEt_3_ to pH = 9.5. HPLC (30 to 60 % B in 15 min): *t*_*R*_ = 14.0 min; ESI-MS: calculated for (C77H106N18O15S2): 1586.8; found: *m/z* = 1587.0 [M+H]^+^.

#### Coupling of NCS-MP-NODA

2,2′-(7-(4-isothiocyanatobenzyl)-1,4,7-triazonane-1,4-diyl)diacetic acid (NCS-MP-NODA) (1.9 mg, 5.4 μmol, 1.5 equiv) was coupled to **A** (3 mg, 3.6 μmol, 1 equiv) with DIPEA (1.1 μL, 3.2 μmol, 2 equiv) in anhydrous DMF to obtain pH ≈ 9. HPLC (20 to 50 % B in 15 min): *t*_*R*_ = 9.0 min; ESI-MS: calculated for (C54H71N13O10S): 1093.5; found: *m/z* = 1095.1 [M+H]+, 1116.7 [M+Na]^+^.

#### Chelation of Ga^3+^ with DOTA, DOTAGA, NOTA, NODAGA conjugates

A solution of Ga(NO_3_)_3_ (250 μL, 2 mM, 1 equiv) in water, pH = 3.0, was added to the chelator-conjugated peptide, (250 μL, 2 mM, 1 equiv) dissolved in H_2_O and, if necessary, in DMSO up to 50 % (*v*/*v*). The final pH of the mixture was adjusted to 4–6 and heated at 90 °C for 30 min. [^nat^Ga]**1**: HPLC (30 to 55 % B in 15 min): *t*_*R*_ = 9.0 min; ESI-MS: calculated for (C60H78GaN14O14): 1287.5; found: *m/z* = 1287.7 [M+H]^+^, 1311.7 [M+Na]^+^; [^nat^Ga]**2**: HPLC (30 to 55 % B in 15 min): *t*_*R*_ = 9.8 min (Ga); ESI-MS: calculated for (C63H82GaN14O16): 1359.5; found: *m/z* = 1361.3 [M+H]^+^, 1383.2 [M+Na]^+^; [^nat^Ga]**3**: HPLC (30 to 55 % B in 15 min): *t*_*R*_ = 9.3 min; ESI-MS: calculated for (C56H71GaN13O12): 1186.5; found: *m/z* = 1188.9 [M+H]^+^, 1209.9 [M+Na]^+^; [^nat^Ga]**4**: HPLC (30 to 55 % B in 15 min): *t*_*R*_ = 10.8 min; ESI-MS: calculated for (C59H74GaN13O14): 1257.5; found: *m/z* = 1258.8 [M+H]^+^.

#### Chelation of In^3+^, Y^3+^, and Lu^3+^ with DOTA, DOTAGA, and DTPA conjugates

A solution of either YCl_3_, LuCl_3_, or InCl_3_ (250 μL, 20 mM, 10 equiv) in ammonium acetate (0.1 M, pH = 6.0) was added to the chelator-conjugated peptide (250 μL, 2 mM, 1 equiv). The pH was adjusted to 4–6, and the mixture heated at 90 °C for 30 min. [^nat^Lu]**1** and [^nat^Y]**1**: HPLC (10 to 50 % B in 15 min): *t*_*R*_ = 10.7 min each; ESI-MS: calculated for (C60H77LuN14O14): 1392.5; found: *m/z* = 1393.8 [M+H]^+^; ESI-MS: calculated for (C60H77N14O14Y): 1306.5; found: *m/z* = 1308.5 [M+H]^+^, 1329.7 [M+Na]^+^; [^nat^Lu]**2** and [^nat^Y]**2**: HPLC (30 to 55 % B in 15 min): *t*_*R*_ = 9.8 min each; ESI-MS: calculated for (C63H80LuN14O16): 1463.5; found: *m/z* = 1465.3 [M+H]^+^, 1488.1 [M+Na]^+^; ESI-MS: calculated for (C63H80N14O16Y): 1377.5; found: *m/z* = 1379.3 [M+H]^+^, 1402.2 [M+Na]^+^; [^nat^In]**7**: HPLC (30 to 55 % B in 15 min): *t*_*R*_ = 10.3 min; ESI-MS: calculated for (C66H77InN14O17S): 1484.4; found: *m/z* = 1487.4 [M+H]^+^; [^nat^Lu]**6**, [^nat^In]**6**, and [^nat^Y]**6**: HPLC (30 to 60 % B in 15 min): *t*_*R*_ = 9.1 min each; ESI-MS: calculated for (C58H71LuN13O16): 1380.5; found: *m/z* = 1382.8 [M+H]^+^, for (C58H71N13O16Y): 1294.4; found: *m/z* = 1296.7 [M+H]^+^, for (C58H71InN13O16): 1320.4; found: *m/z* = 1322.9, [M+H]^+^, 1344.9 [M+Na]^+^.

#### Chelation of Zr^4+^ with **8**

To obtain [^nat^Zr]**8**, a solution of ZrCl_4_ in water (pH = 1.3) (20 mM) was added to the DFO-bearing peptide (2 mM). Quantitative complexation at room temperature occurred within a few minutes without forming any side products. HPLC (30 to 60 % B in 15 min): *t*_*R*_ = 14.0 min; ESI-MS: calculated for (C77H103N18O15S2Zr): 1673.6; found: *m/z* = 1674.5 [M+H]^+^.

#### Chelation of Bi^3+^ with DOTA and DOTAGA conjugates

[^nat^Bi]**1** and [^nat^Bi]**2** were prepared by the dropwise addition of ^nat^Bi(H_3_CCOO)_3_ (10 equiv) to a solution of DOTA or DOTAGA-peptide at pH ~ 5. [^nat^Bi]**1**: HPLC (30 to 55 % B in 15 min): *t*_*R*_ = 8.8 min; ESI-MS: calculated for (C60H77BiN14O14): 1426.6; found: *m/z* = 1427.4 [M+H]^+^, 1451.5 [M+Na]^+^, 1465.1 [M+K]^+^; [^nat^Bi]**2**: HPLC (30 to 55 % B in 15 min): *t*_*R*_ = 9.8 min; ESI-MS: calculated for (C63H80BiN14O16): 1497.6; found: *m/z* = 1499.3 [M+H]^+^, 1521.3 [M+Na]^+^, 1537.3 [M+K]^+^.

#### Chelation of Cu^2+^ with DOTA, DOTAGA, NOTA, NODAGA, and DTPA conjugates

A solution of Cu(OAc)_2_ (250 μL, 2 mM, 1 equiv) in water, pH = 6.0, was added to the chelator-conjugated peptide, (250 μL, 2 mM, 1 equiv). The mixture was incubated at room temperature for 30 min. HPLC revealed quantitative complexation. [^nat^Cu]**1**: HPLC (30 to 55 % B in 15 min): *t*_*R*_ = 10.8 min; ESI-MS: calculated for (C60H77CuN14O14): 1280.5; found: *m/z* = 1282.8 [M+H]^+^, 1306.8 [M+Na]^+^.

[^nat^Cu]**2**: HPLC (30 to 55 % B in 15 min): *t*_*R*_ = 11.0 min; ESI-MS: calculated for (C63H82CuN14O16): 1353.5; found: *m/z* = 1354.8 [M+H]^+^, 1377.8 [M+Na]^+^; [^nat^Cu]**3**: HPLC (30 to 55 % B in 15 min): *t*_*R*_ = 10.5 min; ESI-MS: calculated for (C56H71CuN13O12): 1180.5; found: *m/z* = 1181.8 [M+H]^+^, 1203.6 [M+Na]^+^; [^nat^Cu]**4**: HPLC (30 to 55 % B in 15 min): *t*_*R*_ = 10.9 min; ESI-MS: calculated for (C59H74CuN13O14): 1251.5; found: *m/z* = 1253.8 [M+H]^+^; [^nat^Cu]**6**: HPLC (30 to 55 % B in 15 min): *t*_*R*_ = 9.8 min; ESI-MS: calculated for (C58H72CuN13O16): 1269.5; found: *m/z* = 1271.7 [M+H]^+^, 1232.8 [M+Na]^+^.

#### Chelation of AlF^2+^ with NOTA and NODA conjugates

The peptide was labeled with [^nat^F]AlF by mixing AlCl_3_ (1.2 equiv, 0.468 μmol in 0.5 M NaOAc, pH = 4.0), NaF (1.2 equiv 0.468 μmol in 0.5 M sodium acetate, pH = 4.0) with **3** or **5** (1 equiv, 0.39 μmol). The [^nat^F]AlF-labeled peptide was purified using RP-HPLC on a C_18_ (5 μm, 125 × 4.0 mm). [^nat^F]AlF-**3**: HPLC (23 % B in 15 min): *t*_*R*_ = 10.5 min; ESI-MS: calculated for (C56H71AlFN13O12): 1163.5; found: *m/z* = 1164.9 [M+H]^+^; [^nat^F]AlF-**5**: HPLC (20 to 50 % B in 15 min): *t*_*R*_ = 8.2 min; ESI-MS: calculated for (C54H69AlFN13O10S): 1137.5; found: *m/z* = 1139.0 [M+H]^+^, 1160.9 [M+Na]^+^.

#### Cell culture and determination of CXCR4 receptor affinity (IC_50_)

For in vitro experiments, the Jurkat T - cell line was used. The cells were maintained in RPMI 1640 medium (Biochrom) containing 10 % fetal calf serum (FCS) (Biochrom). The cell line was cultured at 37 °C in a humidified atmosphere with 5 % CO_2_ and passaged two to three times a week, depending on the cell count.

CXCR4 affinities were determined in competitive binding assays using Jurkat cells with [^125^I]FC131 as the radioligand according to a protocol similar to previously published [7]. FC131 (*cyclo(-*d*-Tyr-l-Arg-l-Arg-l-Nal-Gly-*)) [14] was synthesized and iodinated as described previously [7]. Jurkat cells (4 × 10^5^ cells per vial) were incubated with the respective peptide of interest at the final concentrations ranging from 10^−11^ to 10^−5^ M and app. 0.1 nM of [^125^I]FC131. The total sample volume was 250 μL. After an incubation time of 120 min, the vials were centrifuged at 1300 rpm (Heraeus Megafuge, Thermo) for 3 min and the supernatant was removed. The cells were washed twice with 200 μL ice-cold Hank’s balanced salt solution (HBSS). After each washing step, the samples were centrifuged and the supernatant removed. Finally, the amount of displaced and bound radioligand in the combined fractions of the supernatant and the cell pellet was quantified. The half maximal inhibitory concentration (IC_50_) values were determined using GraphPad Prism software.

## Results

Based on the promising results of [^68^Ga]pentixafor [7–11, 15], the influence of different pentixafor-based peptide-linker-chelators on the CXCR4 affinity (IC_50_) has been evaluated. For this purpose, seven different chelators have been coupled via an AMB moiety to the d-Orn side chain of the CPCR4.2 core peptide **A** (Fig. 1): (a) DOTA, (b) DOTAGA, (c) NOTA, (d) 1,4,7-triazacyclononane,1-glutaric acid-4,7-acetic acid (NODAGA), (e) DTPA, (f) p-SCN-Bn-DTPA, (g) p-SCN-Bn-Desferrioxamine (DFO), and (h) p-SCN-Bn-NODA, whereby SCN-Bn-NODA was directly coupled to the pentapeptide core without ABS. Depending on the preferred metals for each chelator, reference Ga^3+^, complexes with AlF^2+^, Zr^4+^, Cu^2+^, In^3+^, Lu^3+^, Y^3+^, and Bi^3+^ were prepared and evaluated in competitive binding assays on Jurkat T - cells with [^125^I]FC131 as the radioactive reference (Table 1).

**Table 1:** IC_50_ values of different metal-chelate conjugates consisting of *cyclo*(-d-Tyr-N-Me-d-Orn(*spacer-[M* ^*3+*^ *]chelator*)-l-Arg-l-2-Nal-Gly-), expressed as mean ± SD (*n* = 3)

Compound	IC_50_/nM	Compound	IC_50_/nM
**1** (DOTA)	102 ± 17	**2** (DOTAGA)	654 ± 263
[^nat^Ga^3+^]**1**	24.8 ± 2.5	[^nat^Ga^3+^]**2**	380 ± 102
[^nat^Bi^3+^]**1**	22.1 ± 7.0	[^nat^Bi^3+^]**2**	29.4 ± 7.6
[^nat^Cu^2+^]**1**	131 ± 11	[^nat^Cu^2+^]**2**	1165 ± 220
[^nat^Lu^3+^]**1**	40.9 ± 12	[^nat^Lu^3+^]**2**	188 ± 2.8
[^nat^Y^3+^]**1**	40.8 ± 27	[^nat^Y^3+^]**2**	126 ± 89
**3** (NOTA)	253 ± 49	**4** (NODAGA)	275 ± 106
[^nat^Ga^3+^]**3**	17.8 ± 7.7	[^nat^Ga^3+^]**4**	342 ± 72
[^nat^Cu^2+^]**3**	46.1 ± 26	[^nat^Cu^2+^]**4**	343 ± 10
[^nat^F]AlF-**3**	220 ± 57		
**5** (SCN-Bn-NODA)	115 ± 24	**6** (DTPA)	53 ± 7.9
[^nat^F]AlF-**5**	329 ± 49	[^nat^Y^3+^]**6**	156 ± 6.7
**7** (SCN-Bn-DTPA)	200 ± 73	[^nat^Lu^3+^]**6**	111 ± 77
[^nat^In^3+^]**7**	74.1 ± 2.4	[^nat^In^3+^]**6**	90 ± 42
**8** (SCN-Bn-DFO)	105 ± 35	[^nat^Cu^2+^]**6**	175 ± 66
[^nat^Zr^4+^]**8**	148 ± 27		

FC131 as control 9.9 ± 2.4 nM

As already demonstrated [7, 8], non-metalated pentixafor (**1**) shows low affinity (IC_50_: 102 ± 17 nM) towards CXCR4, whereas the Lu^3+^ and Y^3+^ complexes (IC_50_: 40.9 ± 12 and 40.8 ± 27 nM, respectively) exhibit affinities quite similar to [^nat^In]pentixafor (IC_50_: 44 ± 4 nM) [7]. Surprisingly, the affinity of the Bi^3+^ complex is even higher (IC_50_: 22.1 ± 7.0 nM) than the affinity of [^nat^Ga]pentixafor (IC_50_: 24.6 ± 2.5 nM) thus making [^213^Bi]pentixafor an attractive α-particle emitting therapeutic analog for endoradiotherapy. The affinity of the corresponding Cu^2+^ complex was low (131 ± 11 nM). With regard to previous reports [7], it is important to notice that absolute IC_50_ values for [^nat^Ga]pentixafor differ because higher cell numbers were used for the IC_50_ experiments in this study (4 × 10^5^ vs 2 × 10^5^ cells/sample).

When switching to the corresponding DOTAGA derivatives, the IC_50_ values indicate a similar order within the series of investigated metal complexes with generally lower affinities (Table 1), except the Bi^3+^-complex, which, again surprisingly, showed only a small decrease of CXCR4 affinity. Thus, with respect to receptor affinity, DOTAGA-pentixafor was found to offer no advantage over DOTA-pentixafor analogs.

A corresponding evaluation of the NOTA and NODAGA derivatives identified [^nat^Ga^3+^]**3** as the ligand with the highest affinity in this study (IC_50_: 17.8 ± 7.7 nM). All other complexes, including the [^nat^F]AlF-NOTA derivative, seem to be unsuitable for further preclinical evaluation or potential clinical application. Similar disappointing results were obtained for NODA-Bn-SCN, DTPA, DTPA-Bn-SCN, and DFO-BN-SCN derivatives.

## Discussion

Experiences in the development of CXCR4-targeting peptides showed that affinities to the CXCR4 receptor can be significantly affected by even moderate structural modifications in the pentapeptide core [16–19], the linker unit [20], or the chelate [7, 8]. This is in contrast to previous experiences of GPCR, glycoprotein, or enzyme-targeting peptides, such as SSTRs, GRPRs (bombesin), and integrins (e.g., αvß_3_, RGD peptides), as well as the prostate-specific membrane antigen (PSMA), to mention only a few. A variety of peptides towards these targets have been developed and—unlike CXCR4—conjugated with a broad spectrum of linker/chelator moieties. For αvß_3_-binding RGD peptides, the Lys side chain of the typically used c(Arg-Gly-Asp-d-Phe-Lys) does not influence the binding of the peptide in the cleft between the two α_v_ and β_3_ subunits that forms the heterodimeric transmembrane glycoprotein [21]. Thus, these RGD peptides tolerate the introduction of spacers and chelator or the formation of multimers, such as dimers, tetramers, and octamers [22, 23]. Similar freedom of variation, although not that multifarious, has been found for SST ligands. Tyr^3^-octreotate for instance was conjugated to both DTPA and DOTA and labeled with ^nat^In, ^nat^Ga, or ^nat^Y. All conjugates, including metal-free octreotate bound hSST2 with high affinity (0.2 to 3.9 nM), regardless of the chelator and metal used [24]. This was confirmed by other SST2 binding peptides, coupled to NODAGA, CB-TE2A, or DOTA and labeled with ^nat^Ga or ^nat^Cu resulting in affinities in a range from 1.3 to 12.5 nM [25]. Moreover, modification of Tyr^3^-octreotate by glucose or cellobiose and 4-[^18^F]fluorobenzaldehyde or 2-fluoropropionic acid resulted in almost unchanged SST2 affinities in the range of 1.3 to 3.1 nM [26]. Hence, compared to CXCR4-binding pentixafor derivatives, where discrepancies varied from 17.6 to 1165 nM (Ga-NOTA and Cu-DOTAGA conjugated to the same highly affine scaffold of CPCR4.2), the effect of chelator and metal exchange was much less pronounced.

Profound investigations on bombesin-receptor mediated imaging agents have shown that bombesin analogs can be conjugated and labeled with a broad range of chelators and metals under retention of their receptor affinity [27]. Smith et al. listed 12 bombesin conjugates, labeled with a variety of metal chelation systems, all of them with an unchanged affinity in the range of 0.5 to 10.5 nM [28, 29].

Regarding PSMA, 14 ^99m^Tc-based imaging agents and five copper compounds were investigated with various common chelators of ^99m^Tc and ^64^Cu, resulting in high affinity for every compound with *K*_i_ values ranging from 0.03 to 16.3 nM [30] and 0.19 to 13.26 nM [31] for Tc and Cu compounds, respectively. Free, Ga^3+^-, and Lu^3+^-labeled PSMA DOTA and DOTAGA conjugates were shown to be highly specific as well, ranging from 10.2 to 54.7 nM [32]. This list can be prolonged with ^18^F-tracers developed by Pomper et al. [33, 34] and other further ^68^Ga-tracers developed by Eder et al. [35, 36].

Although different groups used different models for affinity evaluation, the relative trend shows that, in contrast to the examples above, the CXCR4 affinity is strongly influenced by the entire ligand-spacer-chelator-radiometal construct. During the development of linker-bridged dimers, Demmer et al. could demonstrate that even dimers, consisting of one high affinity and one “non-” CXCR4-binding peptide exhibit higher affinity when compared with the high affinity monomer conjugated with the used linker [20]. The authors conclude a subsite binding of the second peptide unit close to the main binding pocket. Based on the results of this study, we conclude that binding of the AMB-[M^3+^]chelator moiety of [^nat^Ga]pentixafor and [^nat^Ga^3+^]**3** significantly contributes to and is a prerequisite for high affinity binding of the entire peptide ligand. Consequently, depending on the chelator, metalation can have a significant effect on the affinity towards CXCR4.

## Conclusions

In summary, these studies demonstrated that pentixafor, consisting of the cyclic peptide *cyclo(-*d*-Tyr-N-Me-*d*-Orn-l-Arg-l-2-Nal-Gly-)* and conjugated at the Orn side chain with AMB-[^nat^Ga]DOTA, represents a highly optimized ligand. As a result of this study, two further ligands, a Ga-NOTA ([^nat^Ga^3+^]**3**) and a Bi-DOTA ([^nat^Bi^3+^]**1**) derivative with slightly higher affinity to hCXCR4, have been developed. Whereas the Ga^3+^-ligand [^nat^Ga^3+^]**3** suffers from a lower hydrophilicity and thus presumably inferior pharmacokinetics compared to [^nat^Ga]pentixafor, the Bi^3+^-complex is expected to be a very promising new ligand for further studies towards α-emitter-based endoradiotherapeutic approaches, including multiple myeloma and other lymphoproliferative disorders.

## Abbreviations

(NODAGA)(tBu)_3_: 4-(4,7-bis(2-(tert-butoxy)-2-oxoethyl)-1,4,7-triazacyclononane-1-yl)-5(tert-butoxy)-5-oxopentanoic acid
AMB: aminomethylbenzoyl
CXCR4: chemokine receptor 4
DCM: dichloromethane
Dde: N-[1-(4,4-dimethyl-2,6-dioxocyclohexylidene)ethyl]
DIC: N,N′-diisopropyl-carbodiimide
DIPEA: N,N-diisopropylethylamine
DMF: dimethylformamide
DOTA: 1,4,7,10-tetraazacyclododecane-1,4,7,10-tetraacetic acid
DOTAGA: 1,4,7,10-tetraazacyclododecane,1-(glutaric acid)-4,7,10-triacetic acid
DOTAGA-anhydride: 2,2′,2″-(10-(2,6-dioxotetrahydro-2H-pyran-3-yl)-1,4,7,10-tetraazacyclododecane-1,4,7-triyl)triacetic acid
DTPA: diethylenetriaminepentaacetic acid
DTPA(tBu)_4_: 3,6,9-tris(2-(tert-butoxy)-2-oxoethyl)-13,13-dimethyl-11-oxo-12-oxa-3,6,9-triazatetradecan-1-oic acid
EDCI: 1-ethyl-3-(3-dimethylaminopropyl)carbodiimide
FCS: fetal calf serum
Fmoc: fluorenylmethyloxycarbonyl
GRPR: gastrin-releasing peptide receptor
HATU: 1-[bis(dimethylamino)methylene]-1H-1,2,3-triazolo[4,5-b]pyridinium 3-oxid hexafluorophosphate
HBSS: Hank’s balanced salt solution
HOAt: 1-hydroxy-7-azabenzotriazole
HOBt: N-hydroxybenzotriazole
IC_50_: half maximal inhibitory concentration
NCS-MP-NODA: 2,2′-(7-(4-isothiocyanatobenzyl)-1,4,7-triazonane-1,4-diyl)diacetic acid
NHS: N-hydroxysuccinimide
NMP: N-methyl-2-pyrrolidone
NODAGA: 1,4,7-triazacyclononane,1-glutaric acid-4,7-acetic acid
NOTA: 1,4,7-triazacyclononane-triacetic acid
Pbf: 2,2,4,6,7-pentamethyldihydrobenzofuran-5-sulfonyl
Pentixafor: cyclo(-d-Tyr-*N*-Me-d-Orn(AMB-DOTA)-l-Arg-l-2-Nal-Gly-)
PET: positron emission tomography
p-SCN-Bn-DFO: (4-isothiocyanatophenyl)-3-[6,17-dihydroxy-7,10,18,21-tetraoxo-27-(N-acetylhydroxylamino)-6,11,17,22-tetraazaheptaeicosine] thiourea
p-SCN-Bn-DTPA: 2-(4-isothiocyanatobenzyl)-diethylenetriamine pentaacetic acid
PSMA: prostate-specific membrane antigen
SDF-1: stromal cell derived factor-1
SPECT: single photon emission computed tomography
SPPS: solid-phase peptide synthesis
SSTR: somatostatin receptors
TBTU: O-(benzotriazol-1-yl)-N,N,N′,N′-tetramethyluronium tetrafluoroborate
TCP: trityl chloride polystyrene
TFA: trifluoroacetic acid
TIPS: triisopropylsilane
